# Significance of Persistent Inflammation in Patients With Chronic Coronary Syndrome: Insights From the REAL-CAD Study

**DOI:** 10.1016/j.jacadv.2024.100996

**Published:** 2024-06-05

**Authors:** Hiroshi Iwata, Katsumi Miyauchi, Ryo Naito, Satoshi Iimuro, Yukio Ozaki, Ichiro Sakuma, Yoshihisa Nakagawa, Kiyoshi Hibi, Takefui Hiro, Yoshihiro Fukumoto, Seiji Hokimoto, Yasushi Saito, Hisao Ogawa, Hiroaki Shimokawa, Hiroyuki Daida, Takeshi Kimura, Ryozo Nagai

**Affiliations:** a Department of Cardiovascular Biology and Medicine, Juntendo University Graduate School of Medicine, Tokyo, Japan; b Innovation and Research Support Center, International University of Health and Welfare, Tokyo, Japan; c Fujita Health University School of Medicine, Toyoake, Japan; d Caress Sapporo Hokko Memorial Clinic, Sapporo, Japan; e Department of Cardiovascular Medicine, Shiga University of Medical Science Hospital, Otsu, Japan; f Division of Cardiology, Yokohama City University Medical Center, Yokohama, Japan; g Division of Cardiology, Department of Medicine, Nihon University School of Medicine, Tokyo, Japan; h Division of Cardiovascular Medicine, Department of Internal Medicine, Kurume University School of Medicine, Kurume, Japan; i Kumamoto City Ueki Hospital, Kumamoto, Japan; j Chiba University, Chiba, Japan; k Kumamoto University, Kumamoto, Japan; l Department of Cardiovascular Medicine, Tohoku University Graduate School of Medicine, Sendai, Japan; m Division of Cardiology, Hirakata Kohsai Hospital, Osaka, Japan; n Jichi Medical University, Shimotsuke, Japan

**Keywords:** Asians, coronary artery disease, inflammation, outcome, residual risk

## Abstract

**Background:**

The prognostic implications of persistent low-grade inflammation in patients with chronic coronary syndrome (CCS) are underexplored. The REAL-CAD (Randomized Evaluation of Aggressive or Moderate Lipid Lowering Therapy with Pitavastatin in Coronary Artery Disease) study demonstrated the benefit of higher intensity pitavastatin in Japanese patients with CCS.

**Objectives:**

This prespecified subanalysis of the REAL-CAD study aimed to assess the prognostic effect of the persistent low-grade inflammation represented by high-sensitivity C-reactive protein (hs-CRP) in CCS patients.

**Methods:**

The present analysis involved patients without events until 6 months after randomization and whose hs-CRP levels were available at baseline and 6 months (n = 10,460). The primary endpoint was the composite of cardiovascular mortality, myocardial infarction, stroke, and unstable angina hospitalization. Landmark analyses evaluated the prognostic impact of continuous inflammation in 4 groups based on the median levels of hs-CRP (0.5 mg/L for both) at baseline and 6 months. The 4 groups included patient with persistently low, elevated (increased), reduced, and persistently high hs-CRP.

**Results:**

Adjusted Cox proportional hazard analyses demonstrated an increased risk of the primary endpoint in the group with persistently high hs-CRP when compared to the group with persistently low hs-CRP as a reference (adjusted HR: 1.48, 95% CI: 1.18-1.89; *P* = 0.001), but with a similar risk in the group with elevated (HR: 1.07, 95% CI: 0.77-1.49, *P* = 0.68) and reduced (HR: 0.92; 95% CI: 0.66-1.27; *P* = 0.60) hs-CRP.

**Conclusions:**

The study shows that persistent low-grade inflammation is associated with poor outcomes and underscores the need to address residual inflammatory risk in CCS patients. (Randomized Evaluation of Aggressive or Moderate Lipid Lowering Therapy With Pitavastatin in Coronary Artery Disease [REAL-CAD]; NCT01042730)

Lowering of low-density lipoprotein cholesterol (LDL-C) by statins is a firmly established anti-atherosclerotic intervention and is associated with a reduced risk of adverse cardiovascular (CV) events in patients with atherosclerotic CV disease, particularly those with coronary artery disease (CAD).^1^^,^^2^ Previous randomized trials and their metanalyses of intensive vs less-intensive statins have consistently demonstrated a favorable prognostic impact of a more intensive strategy.^3^^,^^4^ Studies of non-statin LDL-C lowering interventions, including ezetimibe and proprotein convertase subtilisin-kexin type 9 (PCSK9) inhibitors, have also showed a significant reduction in the risk of adverse CV events, when they are concomitantly used on the top of statins.^5^^,^^6^ Accordingly, recent guidelines strongly recommend an intensive LDL-C lowering strategy in high-risk patients, particularly in secondary prevention in patients with a history of atherosclerotic CV disease.^7^^,^^8^ However, although these aggressive LDL-C lowering strategies strengthen the notion of “the lower, the better,” there is still intense debate as to whether the positive impact of one or combinations of these LDL-C lowering strategies can be universally explained only by the LDL-C lowering effect.^9^

Previous basic and clinical studies have indicated that statins possess a suppressive effect against vascular inflammation, which is considered to be at the center of the pathophysiology of atherosclerosis,^10^ and such an anti-inflammatory effect by statins has been suggested to be independent of LDL-C lowering properties.^11^ Moreover, the pathophysiological roles of inflammation in atherosclerotic CV disease have been gathering renewed attention, as recent trials have revealed the beneficial prognostic impact of canakinumab and low-dose colchicine, which exert anti-inflammatory effects through their inhibition of key molecules in atherogenesis, interleukin-1 alpha, and NLRP3 inflammasomes, respectively, in patients with a history of acute coronary syndrome and chronic coronary syndrome (CCS).^12^, ^13^, ^14^

The REAL-CAD (Randomized Evaluation of Aggressive or Moderate Lipid Lowering Therapy with Pitavastatin in Coronary Artery Disease) study demonstrated the prognostic superiority of 4 mg/d pitavastatin compared to 1 mg/d in Japanese patients with CCS.^15^ This prespecified subanalysis of the REAL-CAD study aimed to assess the prognostic impact of continuous low-grade inflammation and its possible therapeutic implications.

## Participants and methods

### Study participants and defined outcome measures

REAL-CAD was a prospective randomized open-label trial that enrolled 12,413 patients aged 20 to 80 years old with stable CAD, CCS, and a history of coronary revascularization, including percutaneous coronary intervention and coronary artery bypass graft surgery, or had angiographically proven significant stenosis (>75%) in a coronary artery.^16^ The hypothesis that higher-dose (4 mg/d) as compared with lower-dose (1 mg/d) pitavastatin therapy could reduce CV events was tested in the REAL-CAD study and patients were randomly assigned in a 1:1 ratio to treatment with 4 mg/d or 1 mg/d pitavastatin following a run-in period for at least 1 month. The baseline and follow-up high-sensitivity C-reactive protein (hs-CRP) levels were measured at randomization and at approximately 6 months (184.7 ± 42.1 days) later (6 months follow up hs-CRP). Similar to lipid parameters, such as LDL-C, high-density lipoprotein-cholesterol (HDL-C), and triglycerides, serum was collected and hs-CRP level was centrally measured using a latex-enhanced nephelometry assay (Behring Nephelometer II, Siemens Healthcare Diagnostics Ltd).^17^

For the present subanalysis of the REAL-CAD study, we excluded patients in whom either baseline or follow-up hs-CRP data were not available, and those who had the occurrence of the primary endpoint event before the follow-up hs-CRP measurement (n = 1,953). In the eligible patients for the present analysis (n = 10,460: 84.2% out of the total final analysis set of the REAL-CAD study population), the median levels of baseline and 6-month follow-up hs-CRP were 0.51 mg/L and 0.48 mg/L, respectively. Accordingly, participants in the present analysis were divided into 4 groups based on the cutoff level of hs-CRP (0.5 mg/L) at both baseline and 6-month follow-up; a persistently low hs-CRP group (Group A) (hs-CRP <0.5 mg/L both at baseline and at 6-month follow-up, n = 3,914), reduced hs-CRP group (Group B) (hs-CRP ≥ 0.5 mg/L at baseline and <0.5 mg/L at 6-month follow-up, n = 1,484), elevated hs-CRP group (Group C) (<0.5 mg/L at baseline and ≥0.5 mg/L at 6-month follow-up, n = 1,282), and persistently high hs-CRP group (Group D) (≥0.5 mg/L both at baseline and at 6-month follow-up, n = 3,820) (Supplemental Figure 1). In this study, patients in the persistently high hs-CRP group (Group D) were postulated to have continuous inflammation. The primary outcome measure in the present study was the same as that in the main REAL-CAD study, including the composite of CV death, nonfatal myocardial infarction, nonfatal stroke, and hospitalization for unstable angina. CV death was selected as the secondary outcome measure.^15^^,^^18^

### Statistical analysis

Continuous variables are presented as the mean ± SD or median (IQR) as appropriate. Categorical variables are presented as the actual number and frequencies (%). Quantitative data across groups were compared using the analysis of variance test or the Kruskal-Wallis test. Categorical variables were compared using the Fisher exact test with the chi-squared test. Parametric Pearson correlation analysis was used to evaluate the correlation between the ratio of baseline to follow-up LDL-C (baseline/follow-up) (LDL-C ratio) and logarithmically transformed hs-CRP ratio (log [hs-CRP ratio]) since both the LDL-C ratio and logarithmically transformed hs-CRP ratio were normally distributed. The landmark method was used in the time-to-event analysis, in which the cumulative incidences of primary and secondary outcome measures were assessed and compared 6 months after randomization using unadjusted Kaplan-Meier analysis followed by log-rank comparisons. The HRs with 95% CIs of the 3 hs-CRP groups (Group B-D: reduced hs-CRP group, elevated hs-CRP group, and persistently high hs-CRP group) relative to the reference group (Group A: persistently low hs-CRP group) for the primary and secondary outcome measures were obtained by Cox proportional hazard analyses using a model including the following adjustment factors; covariates, sex, age (65 years or older), current smoker, body mass index (25 or higher), diabetes, atrial fibrillation (AF), chronic kidney disease (CKD) (Grade 3 or 4), and serum levels of lipid parameters at 6-month follow-up, LDL-C, HDL-C, and triglycerides as continuous variables. For the outcome of CV death, we adopted a competing risk framework, in which non-CV death occurring in the absence of the outcome of interest was treated as a competing risk, because the non-CV death precluded the occurrence of the event of interest. We did the modeling with the approach of Fine and Gray,^19^ with effect-sizes presented as subdistribution HRs. All reported *P* values are 2-sided.

## Results

### Baseline characteristics of patients stratified by serum levels of hs-CRP at baseline and 6-month follow-up

Patients in the persistently high hs-CRP group (Group D) were the oldest, while those in the persistently low hs-CRP group (Group A) were the youngest and those in the other 2 groups were in between (Table 1). Patients in the persistently high hs-CRP group (Group D) were more likely to have comorbidities, including hypertension, AF, CKD, and history of heart failure and stroke. The proportion of patients taking beta-blockers and angiotensin-converting enzyme inhibitors/angiotensin receptor blockers was the highest in the persistently high hs-CRP group (Group D) and the lowest in the persistently low hs-CRP group (Group A). The ratios allocated to 4 mg/d of pitavastatin were lower in the groups of high hs-CRP at 6-month follow-up (Group C and D). Collectively, patients in the persistently high hs-CRP group (Group D) had a complex background with more comorbidities, as well as higher intensity medications. Such tendencies and differences were further enhanced when they were compared to those in the persistently low hs-CRP group (Group A). Moreover, baseline and 6-month follow-up levels of hs-CRP were highest in the persistently high hs-CRP group (Group D) and lowest in the persistently low hs-CRP group (Group A). Baseline hs-CRP levels in the reduced hs-CRP group and that of 6-month follow-up in the elevated hs-CRP group were comparable and they had a slightly lower hs-CRP levels compared to persistently high hs-CRP group (Table 2).

**Table 1:** Background Demographics of the 4 Patient Groups Defined by Baseline and 6-Month Follow-Up hs-CRP

	Persistently Low hs-CRP (n = 3,914) (Group A)	Reduced hs-CRP (n = 1,484) (Group B)	Elevated hs-CRP (n = 1,242) (Group C)	Persistently High hs-CRP (n = 3,820) (Group D)	*P* Value
Age (at 6 mo)	67.6 ± 8.5	68.3 ± 8.1	67.7 ± 8.6	68.7 ± 8.0	<0.001
Male	3,205 (81.9%)	1,248 (84.1%)	1,031 (83.0%)	3,187 (83.4%)	0.164
BMI (baseline)	24.0 ± 3.2	24.6 ± 3.1	24.7 ± 3.3	25.4 ± 3.5	<0.001
Current smoking	541 (13.8%)	237 (16.0%)	188 (15.1%)	718 (18.8%)	<0.001
Hypertension	2,881 (73.6%)	1,108 (74.7%)	938 (75.5%)	3,038 (79.5%)	<0.001
Diabetes	1,488 (38.0%)	579 (39.0%)	548 (44.1%)	1,568 (41.0%)	0.001
HbA1c, mg/dL (at 6 mo)	5.8 ± 0.8	5.8 ± 0.8	5.9 ± 0.8	5.9 ± 0.9	<0.001
Atrial fibrillation	190 (4.9%)	91 (6.1%)	77 (6.2%)	286 (7.5%)	<0.001
Chronic kidney disease	1,219 (31.6%)	516 (35.3%)	405 (33.1%)	1,546 (41.0%)	<0.001
eGFR, L/min/1.73 m^2^ (baseline)	67.4 ± 16.2	66.3 ± 15.8	67.0 ± 25.3	63.8 ± 18.0	<0.001
History of myocardial infarction	1,992 (50.9%)	777 (52.4%)	690 (55.6%)	1,964 (51.4%)	0.003
History of heart failure	159 (4.1%)	70 (4.7%)	58 (4.7%)	225 (5.9%)	0.003
History of stroke	263 (6.7%)	114 (7.7%)	90 (7.2%)	348 (9.1%)	0.001
History of malignancy	207 (5.3%)	83 (5.6%)	58 (4.7%)	215 (5.6%)	0.596
Beta-blockers	1,461 (39.7%)	556 (40.3%)	483 (41.7%)	1,587 (44.3%)	0.001
ACEI/ARB	2,377 (64.6%)	938 (68.0%)	807 (69.6%)	2,500 (69.8%)	<0.001
DAPT	1,596 (43.4%)	606 (43.9%)	500 (43.1%)	1,626 (45.4%)	0.31
Allocated to pitavastatin 4 mg	1,944 (50.1%)	719 (53.0%)	609 (45.0%)	1,765 (45.6%)	<0.001

Values are mean ± SD or n (%).

ACEI/ARB = angiotensin-converting enzyme inhibitors/angiotensin receptor blockers; BMI = body mass index; DAPT = dual antiplatelet therapy; eGFR = estimated glomerular filtration rate; HbA1c = hemoglobin A1c; hs-CRP = high-sensitivity C-reactive protein.

**Table 2:** Temporal Changes in hs-CRP and Lipid Parameters in the 4 Groups

	Persistently Low hs-CRP (n = 3,914) (Group A)	Reduced hs-CRP (n = 1,484) (Group B)	Elevated hs-CRP (n = 1,242) (Group C)	Persistently High hs-CRP (n = 3,820) (Group D)	*P* Value
hs-CRP					
Baseline	0.22 (0.13-0.33)	0.85 (0.63-1.59)	0.32 (0.22-0.41)	1.28 (0.81-2.45)	<0.001
6 mo	0.20 (0.12-0.31)	0.32 (0.22-0.42)	0.87 (0.64-1.78)	1.21 (0.76-2.37)	<0.001
LDL-C					
Baseline	87.9 ± 18.7	87.0 ± 18.8	87.2 ± 19.1	87.9 ± 18.9	0.253
6 mo	81.1 ± 20.9	79.4 ± 21.1	81.6 ± 21.8	81.2 ± 22.2	0.03
12 mo	82.2 ± 21.2	82.2 ± 21.2	83.1 ± 21.8	83.9 ± 22.1	0.004
24 mo	83.1 ± 21.5	82.2 ± 21.8	83.1 ± 21.7	84.3 ± 22.9	0.021
36 mo	83.5 ± 22.0	81.7 ± 21.9	84.7 ± 25.2	84.1 ± 23.0	0.014
HDL-C					
Baseline	53.1 ± 12.8	50.2 ± 12.3	51.0 ± 11.8	48.2 ± 11.7	<0.001
6 mo	53.2 ± 12.7	51.4 ± 12.3	50.0 ± 12.0	48.2 ± 11.9	<0.001
12 mo	54.1 ± 14.0	52.0 ± 13.0	51.9 ± 13.5	49.3 ± 13.2	<0.001
24 mo	54.1 ± 13.8	52.2 ± 13.3	51.8 ± 13.6	49.6 ± 12.8	<0.001
36 mo	54.4 ± 14.1	52.4 ± 13.5	51.6 ± 12.6	49.7 ± 13.5	<0.001
Triglycerides					
Baseline	114 (83-161)	118 (84-166)	123 (89-174)	137 (100-194)	<0.001
6 mo	109 (79-152)	118 (85-165)	112 (82-159)	131 (96-186)	<0.001
12 mo	105 (77-146)	115 (81-158)	116 (83-165)	129 (93-182)	<0.001
24 mo	106 (77-150)	113 (81-162)	116 (82-162)	129 (92-181)	<0.001
36 mo	104 (76-146)	113 (81-160)	114 (82-164)	130 (92-181)	<0.001

Values are median (IQR) or mean ± SD.

HDL-C = high-density lipoprotein cholesterol; hs-CRP = high-sensitivity C-reactive protein; LDL-C = low-density lipoprotein cholesterol.

Lipid parameters at multiple time points according to groups are listed and compared in Table 2. In the persistently high hs-CRP group (Group D), the levels of serum LDL-C and triglycerides were also constantly higher, and HDL-C was lower than those in patients with reduced and persistently low hs-CRP (Group A and B), partially due to the lower proportion of those assigned to 4 mg/d pitavastatin. However, *P* values in these comparisons are needed to be interpreted with caution, as they are not adjusted for multiple comparisons.

**Figure 1:**
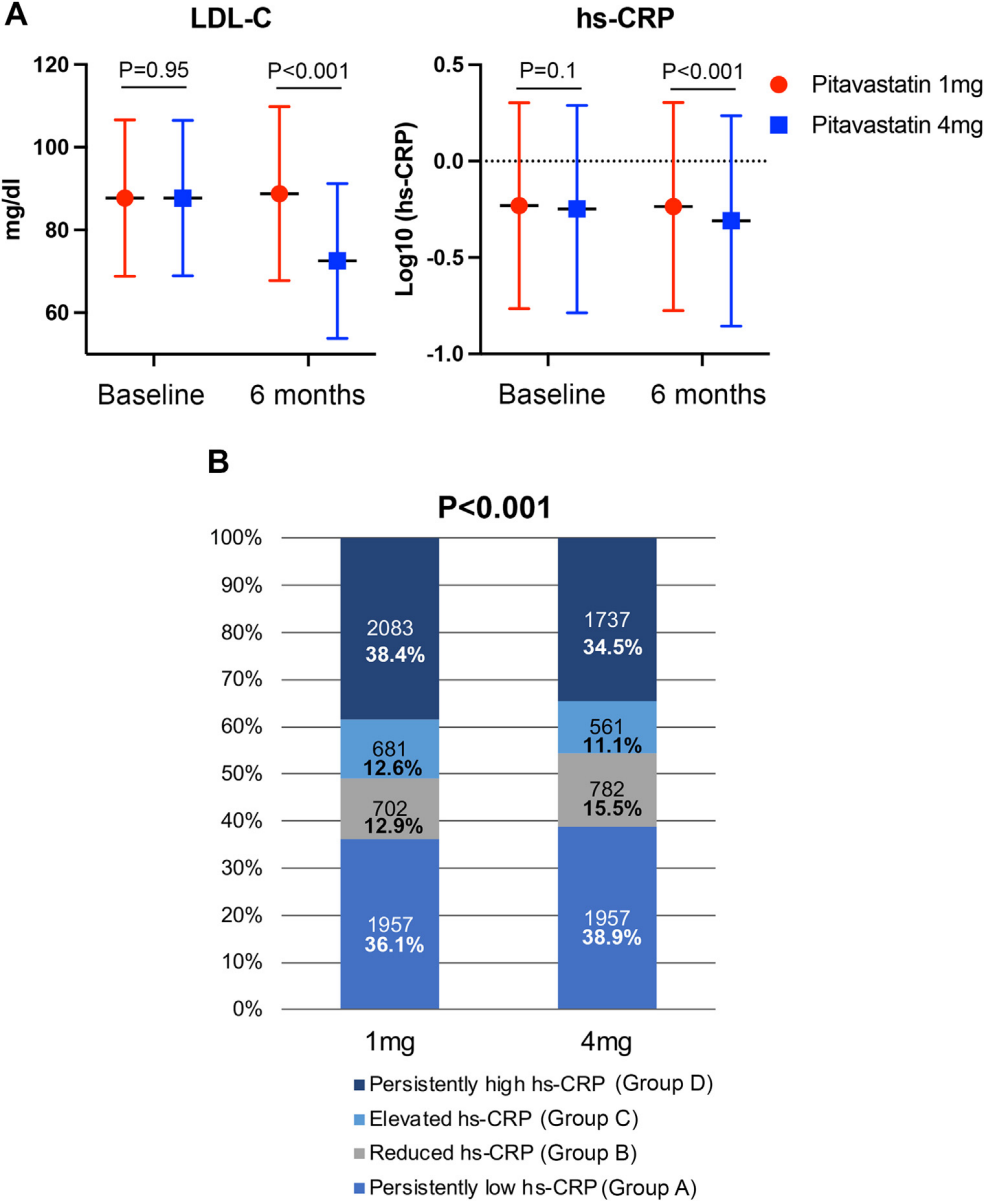
Temporal Changes in LDL-C and hs-CRP, and Distributions of 4 Groups Defined by hs-CRP Change From Baseline to 6 Months in Pitavastatin 1 mg/d vs 4 mg/d Groups (A) Averages ± SDs of LDL-C levels and logarithmically transformed hs-CRP in patients assigned to 1 mg/d vs 4 mg/d pitavastatin. (B) Distributions of 4 groups regarding inflammation at baseline and 6-month follow-up in patients assigned to 1 mg/d vs 4 mg/d pitavastatin. hs-CRP = high-sensitivity C-reactive protein; LDL-C = low-density lipoprotein cholesterol.

The level of hs-CRP at 6-month follow-up was significantly lower in patients allocated to 4 mg/d pitavastatin than in those allocated to 1 mg/d pitavastatin, although they were similar at baseline (Figure 1A). Moreover, by randomized allocations, the proportion in the persistently high hs-CRP group (Group D) was significantly lower in those allocated to 4 mg/d (34.5%) than in those allocated to 1 mg/d pitavastatin (38.4%). In contrast, that of the persistently low hs-CRP group (Group A) was higher in patients with 4 mg/d compared to 1 mg/d pitavastatin (38.9% vs 36.1%, respectively) (*P* < 0.001) (Figure 1B).

### Clinical outcomes

**Figure 2:**
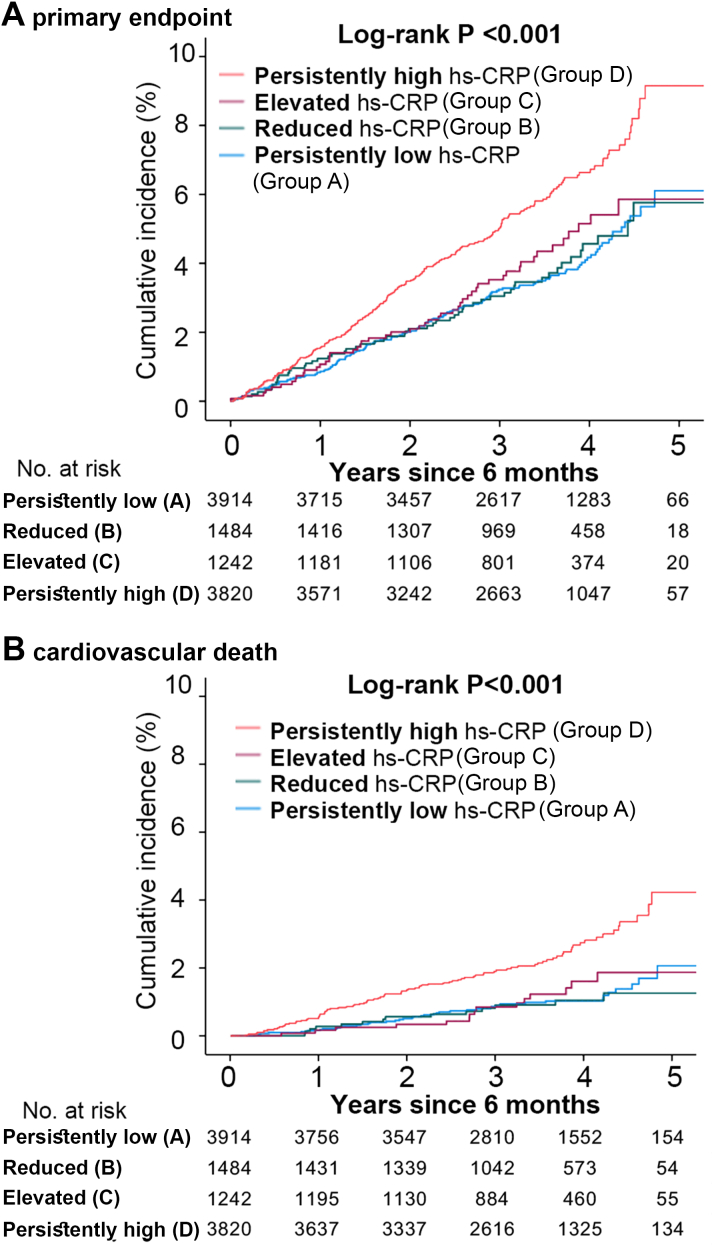
Landmark Analysis Starting From 6 Months After Randomization in Patients Stratified by Change of hs-CRP Landmark analysis of cumulative incidences of primary endpoint (A) and cardiovascular death (B) in 4 patient groups; persistently low hs-CRP (hs-CRP <0.5 mg/L at baseline and 6 months), reduced hs-CRP (hs-CRP >0.5 mg/L at baseline, and ≤0.5 mg/L at 6 months), elevated hs-CRP (hs-CRP ≤0.5 mg/L at baseline and 0.5 mg/L at 6 months), and persistently high hs-CRP (hs-CRP >0.5 mg/L at baseline and 6 months).

**Figure 3:**
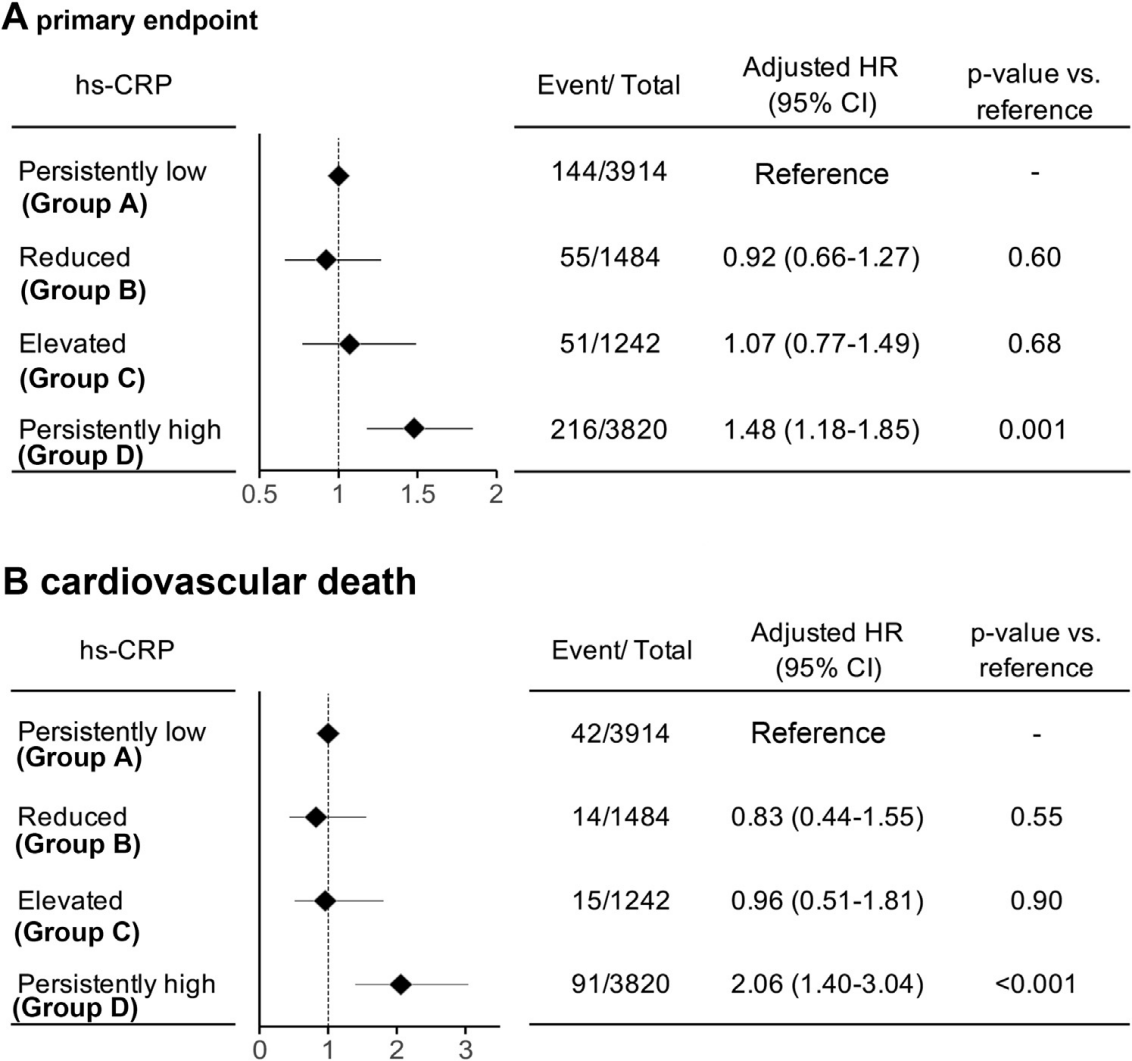
Adjusted Risks of Persistently High hs-CRP for Outcomes Adjusted risks of persistently high hs-CRP for primary endpoint (A) and cardiovascular mortality (B).

Median follow-up durations in this study were 3.3 and 3.5 years after 6-month follow-up for the primary and secondary outcome measures, respectively. Landmark unadjusted Kaplan-Meier analysis since follow-up hs-CRP measurement at 6 months demonstrated a significantly higher cumulative incidence of the primary outcome measure in the group with persistently high hs-CRP (Group D), compared to other 3 (Figure 2A). Similarly, the cumulative incidence of CV death, which is the secondary outcome measure of the present study, was substantially higher in the persistently high hs-CRP group (Group D) than in other 3 groups (Group A to C) (Figure 2B). In these 3 groups, the cumulative incidences of the primary and secondary outcome measures were similar. Even after adjusting for the confounders, including sex, age, current smoker, body mass index, diabetes, AF, CKD, and serum levels of lipid parameters at 6-month follow-up, LDL-C, HDL-C, and triglycerides, the excess risk of the persistently high hs-CRP group (Group D) relative to the persistently low hs-CRP group (Group A) remained highly significant for both the primary and secondary outcome measures, while there was no excess risk of the reduced and elevated hs-CRP groups (Group B and C) relative to the persistently low hs-CRP group (Group A) for the primary and secondary outcome measures (Figure 3A and B). For the CV death, subdistribution HRs were 1.60 (95% CI: 1.01-2.54, *P* = 0.048) for Group D, 1.42 (95% CI: 0.89-2.26, *P* = 0.14) for Group C, and 0.79 (95% CI: 0.46-1.36, *P* = 0.39) for Group B, respectively (Group A served as reference) (Supplemental Table 1).

**Figure 4:**
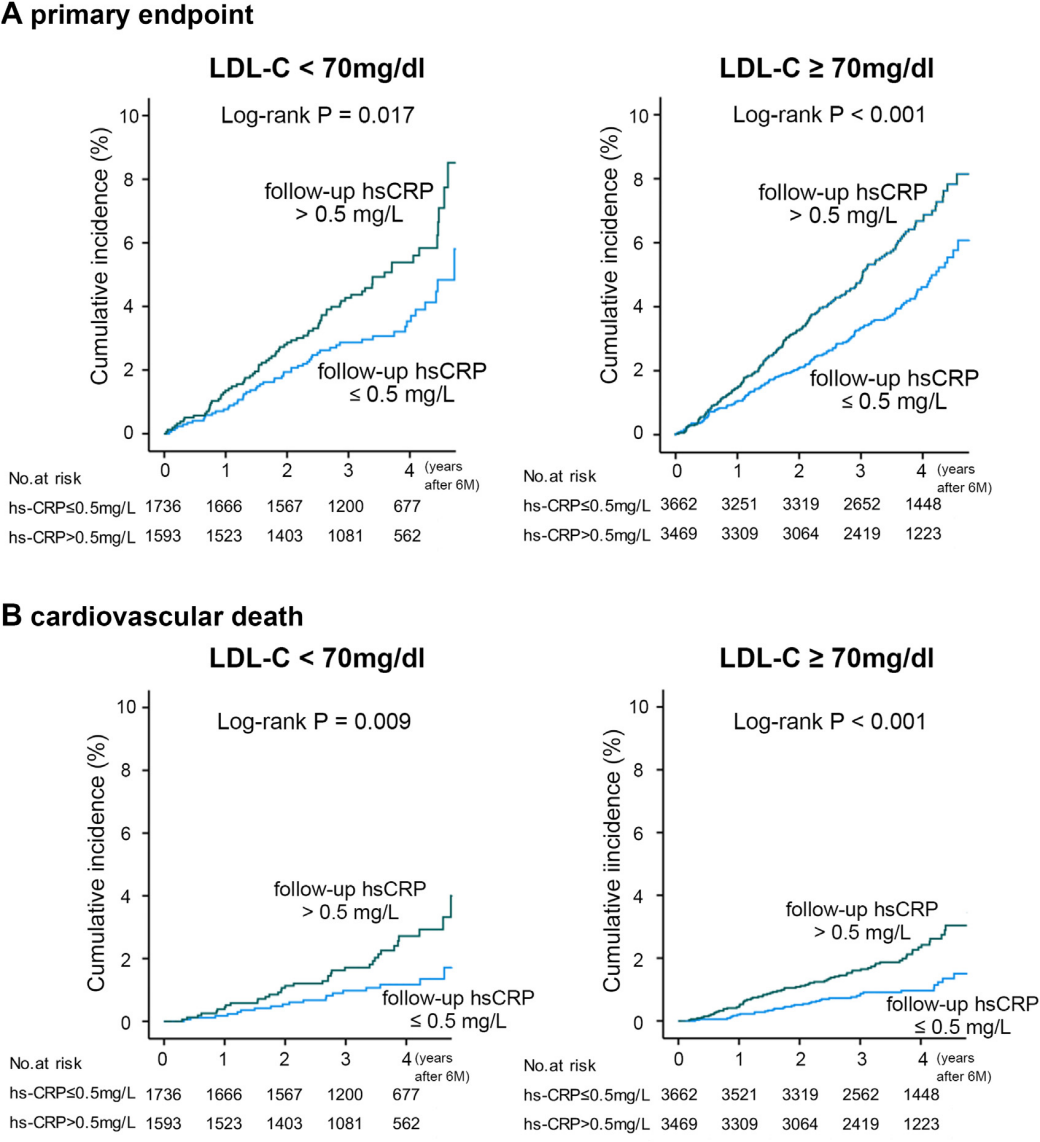
Prognostic Impact of hs-CRP at 6 Months in Patients With and Without Controlled LDL-C (<70 and ≥70 mg/dL) Landmark Kaplan-Meier curves of cumulative incidences of primary endpoint (A) and cardiovascular mortality (B) in patients with hs-CRP >6 and ≤ at 6 months whose LDL-C was controlled to be lower than 70 mg/dL at 6 months (green) or not (blue) irrespective of REAL-CAD study assignment. hs-CRP = high-sensitivity C-reactive protein; LDL-C = low-density lipoprotein cholesterol.

To assess the effect of inflammation in patients with and without achieving LDL-C goals in guideline-directed lipid-lowering therapy (<70 mg/dL)^7^^,^^8^^,^^20^ irrespective of randomized allocation, landmark Kaplan-Meier analyses stratified by the level of hs-CRP at 6 months (>0.5 vs ≤0.5 md/L) were performed in patients with LDL-C ≥70 and <70 mg/dL. As a result, the differences in the cumulative incidences of the composite primary and secondary outcome measures between patients with and without higher hs-CRP were consistently significant even in those who achieved goals of LDL-C (<70 mg/dL) (Figures 4A and 4B). Moreover, nonparametric Spearman correlation analysis between the difference in LDL-C and hs-CRP from baseline to 6 months after randomization showed very weak correlation in the entire participant population and no correlation in those who were assigned to 1 mg/d and 4 mg/d of pitavastatin in the REAL-CAD study (Supplemental Figures 2A to 2C).

## Discussion

### Outcomes

The results of this prespecified subanalysis of the REAL-CAD study indicated a significant association between the presence of persistent inflammation and poor outcomes. Importantly, such a negative prognostic impact due to persistent inflammation was independent from baseline and achieved levels of LDL-C by pitavastatin (Central Illustration).

**Central Illustration:**
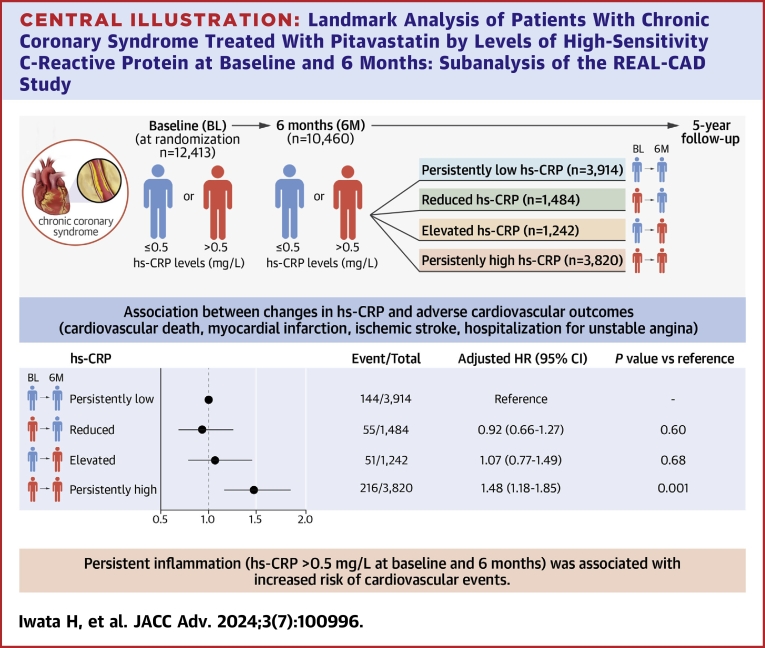
Landmark Analysis of Patients With Chronic Coronary Syndrome Treated With Pitavastatin by Levels of High-Sensitivity C-Reactive Protein at Baseline and 6 Months: Subanalysis of the REAL-CAD Study hs-CRP = high-sensitivity C-reactive protein; CCS = chronic coronary syndrome.

To reduce the risk of atherosclerotic CV disease, particularly in secondary prevention for patients with coronary, cerebrovascular, and peripheral atherosclerotic diseases, the current guidelines recommend intensive LDL-C lowering strategies using a combination of statins with ezetimibe or PCSK9 inhibitors.^7^^,^^8^ Particularly, previous intensive versus less-intensive statin outcome trials including REAL-CAD have suggested significant superiority for higher intensity statin therapy for reducing the risk of CV events as compared with less-intensive statin therapy.^3^^,^^4^^,^^15^ Furthermore, non-statin LDL-C lowering medications in conjugation with statins, ezetimibe and PCSK9 inhibitors have also been shown to have benefits for further reduction in CV event risk compared to statins alone.^5^^,^^6^ However, despite the significant reduction in LDL-C levels by the combination of statins and non-statin medications, a substantial number of patients are still at risk for adverse CV events.^5^^,^^6^ Moreover, the prognostic benefit by LDL-C lowering medications was not completely parallel to the extent of LDL-C lowering. Indeed, a meta-analysis demonstrated that the risk reduction was more prone by increasing the intensity of statins compared to the addition of ezetimibe and PSCK-9 inhibitors, when it was adjusted by the extent of LDL-C reduction.^9^ This evidence supports the hypothesis that statins, as well as ezetimibe, possess beneficial pleotropic effects other than LDL lowering, which was not obvious for PCSK9 inhibitors.^21^, ^22^, ^23^ Chronic vascular inflammation plays crucial roles in the pathogenesis of atherosclerosis, which is regarded as a complex pathological process. Previous observational studies have indicated a significant pathological link between chronic inflammation and poor CV outcomes.^16^

Pharmaceutical interventions targeting inflammation, which is independent from lipid lowering, have recently been shown to reduce CV risk.^12^, ^13^, ^14^ CANTOS (Canakinumab Antiinflammatory Thrombosis Outcome Study) was the first to provide a proof-of-concept for the inflammatory hypothesis, demonstrating that the cumulative incidence of CV events in patients with a history of myocardial infarction and elevated level of hs-CRP (2 mg/L or higher) was significantly lower in patients with interleukin-1b neutralizing antibody canakinumab than in those without it^12^ More recently, the COLCOT (Colchicine Cardiovascular Outcomes Trial) and LODOCO2 (Low Dose Colchicine Trial) trials demonstrated a substantial risk reduction by low-dose colchicine in patients with acute coronary syndrome and CCS.^13^^,^^14^ These interventions have shown promise in reducing inflammation-driven risk independently of changes in lipid parameters. These studies in turn reprovoked the discussion regarding clinical significance of the inflammation-centered risk in atherosclerotic CV disease, residual inflammatory risk, even in individuals receiving sufficient contemporary secondary prevention management.^24^^,^^25^

This subanalysis of the REAL-CAD study has demonstrated a significant association between the presence of persistent inflammation and adverse CV outcomes in patients with CCS who were treated by low or moderate intensity pitavastatin, independent from known risk factors, particularly LDL-C levels, for patients with CCS. In the previous studies that evaluated the effects of treatments targeting inflammation on outcomes of atherosclerotic CAD, the medians of baseline hs-CRP levels were at least 2 mg/L,^4^^,^^11^, ^12^, ^13^, ^14^ which were more than 4 times higher than in the present study. Therefore, the present study provides novel evidence that the residual inflammatory risk is significant even in a population whose inflammatory level was substantially lower than in the previous studies. Moreover, these findings are consistent with the previous cohort studies indicating a possible diversity in the levels of inflammation and hs-CRP by race and ethnicity.^26^, ^27^, ^28^ A recent health care-based Swedish study of patients after myocardial infarction showed that elevated hs-CRP >2 mg/L was seen in more than 60%, and indicated linear associations between levels of hs-CRP and CV risks, when they were within 2 to 5 mg/L, and that association was not shown in <1 mg/L.^29^ Therefore, the prognostic impact of hs-CRP <1 mg/L has not been clarified.

A number of basic and preclinical studies have explored the anti-inflammatory effects of statins, ezetimibe, and PSCK9 inhibitors other than their LDL-C lowering properties. Statins, in particular, have been extensively studied and found to have a pleiotropic anti-inflammatory property by their modulation of key players in innate and adaptive immunity involving atherogenesis, such as inflammatory cytokines, such as interleukin-6 and tumor necrosis factor-alpha, metalloproteinases, transforming growth factor-beta signaling, and reactive oxygen species.^30^, ^31^, ^32^ Moreover, previous experimental and clinical studies have also indicated that ezetimibe has a similar anti-inflammatory effect.^33^^,^^34^ In this study, the proportion of persistently high hs-CRP was significantly lower, while that of persistently low hs-CRP was higher in patients who were allocated to pitavastatin 4 mg/d compared to 1 mg/d, indicating the enhanced potency of the anti-inflammatory property of a higher dose of pitavastatin. These results were in line with the findings of the previous statin trials, whereby more intensive statin therapy was associated with reduced hs-CRP.^3^^,^^4^^,^^12^, ^13^, ^14^^,^^35^ The combination of statin and ezetimibe yielded similar results,^36^ while trials of PCSK9 inhibitors and their metanalysis have not shown a substantial impact on hs-CRP despite their potent LDL-C lowering property.^22^^,^^36^^,^^37^ Moreover, consistent with previous investigations, no correlation was observed in the extent of LDL-C and hs-CRP lowering in the entire participant population of the REAL-CAD study and those assigned to receive 1 mg/d and 4 mg/d pitavastatin. These results indicate that the mechanisms of LDL-C lowering and anti-inflammatory property of pitavastatin are not dependent each other.

### Study limitations

Several limitations of the present study should be considered. First, when evaluating the outcomes by hs-CRP level at randomization and 6 months later, the patient groups were not randomized. Therefore, the results of this study need to be interpreted with caution because they might have been influenced by unknown confounding factors and they do not necessarily indicate a causal relationship, although the prognostic impact was adjusted by multiple covariates. Furthermore, given the significant differences in the incidence of comorbidities among the groups, there is a potential risk of statistical bias due to inadequacy in the adjustment by the multivariate model. Second, due to the very low levels of hs-CRP in the REAL-CAD study population, it was difficult to assess the impact of the temporal change in hs-CRP (absolute difference or the ratio between baseline and 6-month follow-up). Third, the lack of data on additional inflammatory markers other than hs-CRP, such as interleukin-6, tumor necrosis factor-alpha, and molecules in transforming growth factor beta signaling might be a limitation of this study. The absence of these markers may restrict a comprehensive understanding of the inflammatory profile in this study population. Furthermore, as magnetic resonance imaging could provide insights into macrophage activation and its association with inflammation in atherosclerotic lesions,^38^ future studies incorporating magnetic resonance imaging may provide a more comprehensive assessment of the inflammatory burden in patients with CCS. Despite these shortcomings, the present findings in the subanalysis of the REAL-CAD study have further strengthen the inflammation hypothesis in atherosclerosis by evaluating the prognostic impact of the continuous inflammation in CCS patients based on rigorous measurement of hs-CRP at 2 time points with a 6-month interval. Furthermore, the findings in this study indicate that persistent inflammation is an important therapeutic target when attempting to reduce the residual risk of atherosclerotic CV disease.

## Conclusions

The present subanalysis of the REAL-CAD study indicate that persistent inflammation is substantially associated with poor outcomes even in Japanese CCS patients taking pitavastatin, although the levels of hs-CRP in this population are relatively low. Present findings may emphasize the need for a comprehensive approach that considers both achieved LDL-C levels and residual inflammatory risk in order to optimize patient management.

PERSPECTIVES **COMPETENCY IN MEDICAL KNOWLEDGE:** Critical roles of residual inflammation risk in atherosclerotic CV disease are gathering renewed attention. However, prognostic implications of low-grade inflammation in patients with CCS remain underexplored. In the REALCAD study, Japanese CCS patients exhibited lower inflammation levels than in previous studies.

**TRANSLATIONAL OUTLOOK:** This subanalysis of the REAL-CAD study showed the adverse prognostic impact of low-grade persistent inflammation (hs-CRP >0.5 mg/l). These findings underscore the need for comprehensive approaches considering residual inflammatory risk in CCS patients.

## Funding support and author disclosures

The Comprehensive Support Project for Clinical Research of Lifestyle-Related Disease of the Public Health Research Foundation funded this study. The company manufacturing the study drug (Kowa Co Ltd) was one of the entities providing financial support for Public Health Research Foundation projects but was not involved in design, analysis, data interpretation, or manuscript preparation. Dr Iwata has received payment or honoraria for lectures, presentations, Speakers Bureaus from Bayer Yakuhin, Ltd, MSD KK, Kowa Co Ltd, Mitsubishi Tanabe Pharma Corp, Sumitomo Pharma, and Mochida Pharmaceutical Co, Ltd. Dr Miyauchi has received payment or honoraria for lectures, presentations, Speakers Bureaus, manuscript writing, or educational events from AstraZeneca KK, Bayer Yakuhin, Ltd, Bristol-Myers Squibb, Daiichi-Sankyo Co Ltd, Kowa Co Ltd. Dr Iimuro has received all support for the present manuscript (data management fee) from Public Health Research Foundation (REAL-CAD office) to Innovation and Research Support Center, International University of Health and Welfare. Dr Sakuma has received grants or contracts from any entity from Public Health Research Foundation from National Cerebral and Cardiovascular Center, Medical Informatics Study Group, GlaxoSmithKline KK, AstraZeneca KK, Takeda Pharmaceutical Co Ltd, and Boehringer lngelheim Japan Inc. Dr Nakagawa has received payment or honoraria for lectures, presentations, Speakers Bureaus, manuscript writing, or educational events from Kowa Co Ltd and Daiichi Sankyo Co, Ltd. Dr Fukumoto has received royalties or licenses from Daiichi Sankyo Co, Ltd, Otsuka Pharmaceutical Co, Ltd, Teijin Pharma Ltd, Bayer Yakuhin, Ltd, Mochida Pharmaceutical Co, Ltd, Astellas Pharma Inc, Sanwa Kagaku Kenkyusho Co, Ltd, Mitsubishi Tanabe Pharma Corp, Takeda Pharmaceutical Co Ltd, Pfizer Japan Inc, Ono Pharmaceutical Co Ltd, and AstraZeneca KK; and payment or honoraria for lectures, presentations, Speakers Bureaus, manuscript writing, or educational events from AstraZeneca KK, Eisai Co, Ltd, MSD KK, Otsuka Pharmaceutical Co, Ltd, Daiichi Sankyo Co, Ltd, Sumitomo Dainippon Pharma Co, Ltd, Teijin Pharma Ltd, Astellas Pharma Inc, Sanwa Kagaku Kenkyusho Co, Ltd, Mitsubishi Tanabe Pharma Corp, Takeda Pharmaceutical Co Ltd, Pfizer Japan Inc, Ono Pharmaceutical Co Ltd, and AstraZeneca KK. Dr Ogawa has received payment or honoraria for lectures, presentations, Speakers Bureaus, manuscript writing, or educational events from Bayer Yakuhin. Dr Daida has received payment or honoraria for lectures, presentations, Speakers Bureaus, manuscript writing, or educational events from Novartis Pharma K.K., Bayer Yakuhin, Ltd, Sanofi KK, Kowa Co Ltd, Taisho Pharmaceutical Co, Ltd, Abbott Medical Japan LLC, Otsuka Pharmaceutical Co, Ltd, Amgen KK, MSD KK, Daiichi Sankyo Co Ltd, Pfizer Japan Inc, Fukuda Denshi Co Ltd, Tsumura & Co, TOA EIYO LTD; and grants or contracts from any entity (trust research/joint research funds/scholarship fund) from Philips Japan, Ltd, FUJIFILM Holdings Co, Asahi Kasei Co, Inter Reha Co, Ltd, TOHO HOLDINGS Co Ltd, GLORY LTD, BMS KK Abbott Japan LLC, Boehringer Ingelheim Japan, Inc, Eisai Co Ltd, Bayer Yakuhin Ltd, and Daiichi Sankyo Co, Ltd. Dr Nagai has received payment or honoraria for lectures, presentations, Speakers Bureaus, manuscript writing, or educational events from Kowa Co Ltd and Shionogi & Co Ltd. All other authors have reported that they have no relationships relevant to the contents of this paper to disclose.
