# Supplementary material for: Significance of Persistent Inflammation in Patients With Chronic Coronary Syndrome: Insights From the REAL-CAD Study
Source: JACC Adv. 2024 Jun 5;3(7):100996. doi: 10.1016/j.jacadv.2024.100996 (PMC11312795; doi:10.1016/j.jacadv.2024.100996)
Supplement: Supplemental Figures 1 and 2, and Table 1 [file mmc1.docx]

**Supplemental Figure 1: A Study scheme**


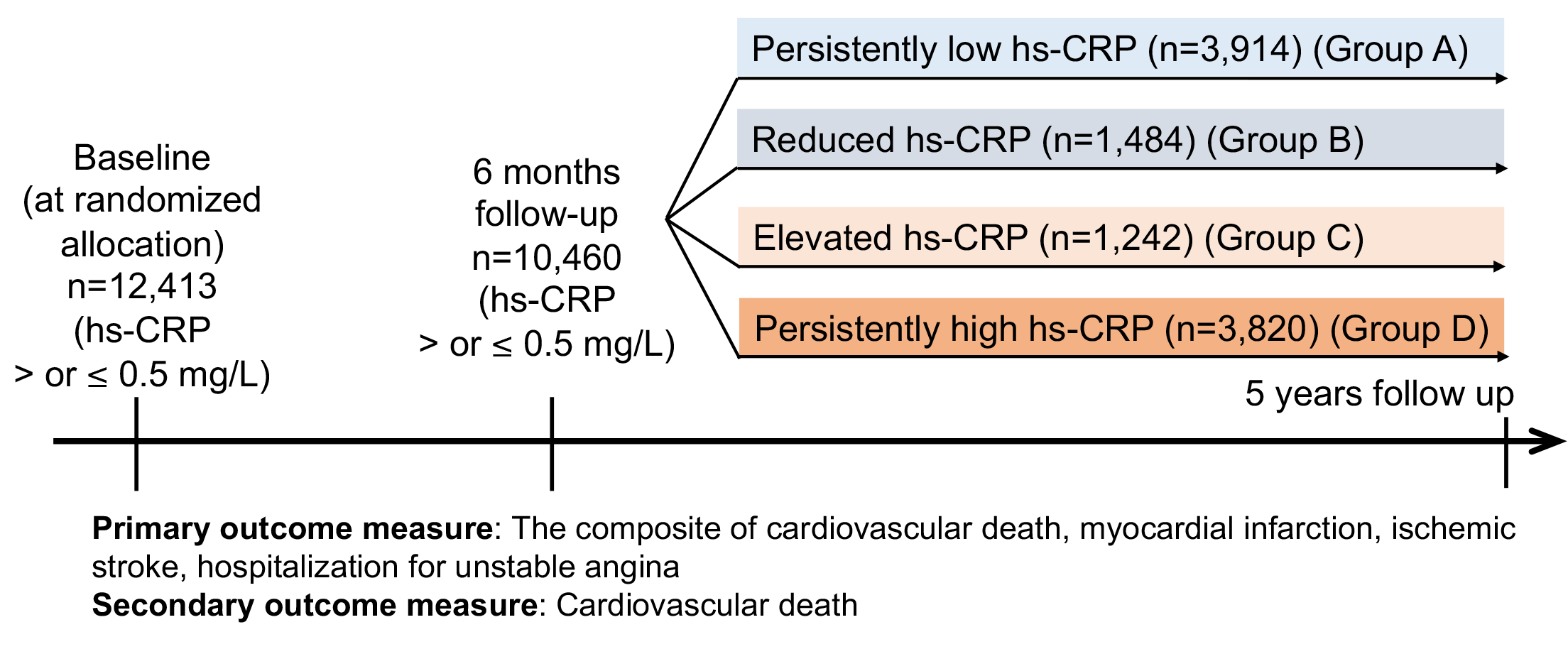


**Supplemental Figure 2: Correlation between the changes in LDL-C and hs-CRP from baseline to 6 months follow-up**


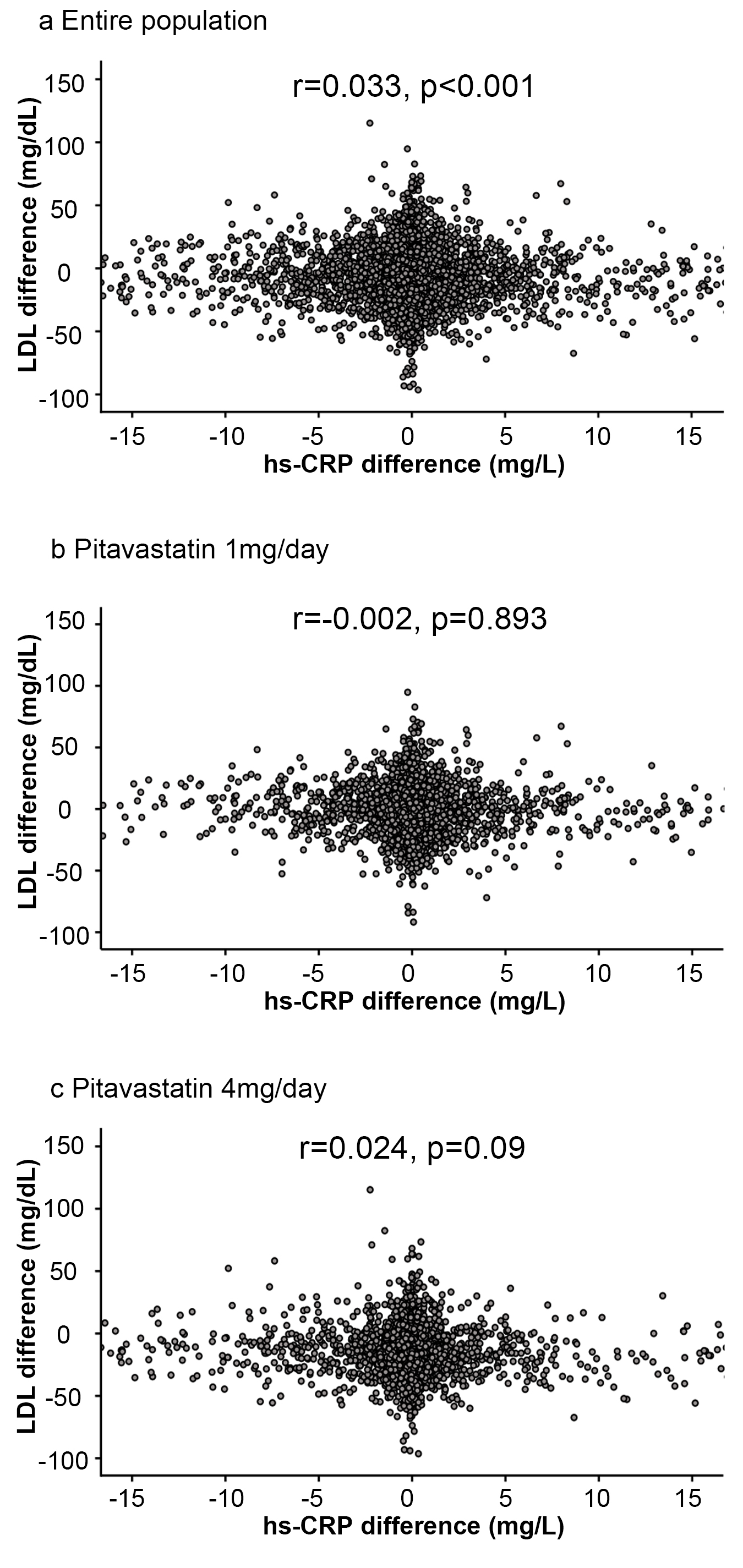


Spearman correlation analysis between the LDL-C difference (6 months minus baseline) and that of hs-CRP ratio in entire population (**a**), patients assigned to pitavastatin 1 mg/day (**b**) and those to 4 mg/day (**c**). r: correlation coefficient

**Supplemental Table 1: Subdistribution HR for the cardiovascular death**

|  | Subdistribution HR | 95% confidence interval | p-value |
| --- | --- | --- | --- |
| Persistently low hs-CRP (Group A) | Reference |  |  |
| Reduced hs-CRP (Group B) | 0.79 | 0.46-1.36 | 0.39 |
| Elevated hs-CRP (Group C) | 1.42 | 0.89-2.26 | 0.14 |
| Persistently high hs-CRP (Group D) | 1.60 | 1.01-2.54 | 0.048 |
| Older than 65 years old | 2.07 | 1.32-3.27 | 0.002 |
| Sex, male | 1.54 | 0.5-3.27 | 0.08 |
| Body mass index >25 | 1.08 | 0.78-1.50 | 0.66 |
| Atrial fibrillation | 1.69 | 1.04-2.74 | 0.03 |
| Smoking habit | 0.92 | 0.57-1.48 | 0.73 |
| Diabetes | 1.79 | 1.31-2.46 | <0.001 |
| Chronic kidney disease | 2.06 | 1.48-2.86 | <0.001 |
| LDL-C at 6 months (1mg/dl) | 1.00 | 0.98-1.01 | 0.16 |
| HDL-C at 6 months (1mg/dl) | 1.00 | 1.00-1.01 | 0.91 |
| Triglycerides at 6 months (1mg/dl) | 1.00 | 1.00-1.00 | 0.26 |
